# Tramadol and Propentofylline Coadministration Exerted Synergistic Effects on Rat Spinal Nerve Ligation-Induced Neuropathic Pain

**DOI:** 10.1371/journal.pone.0072943

**Published:** 2013-08-29

**Authors:** Jin Zhang, Dan Wu, Cheng Xie, Huan Wang, Wei Wang, Hui Zhang, Rui Liu, Li-Xian Xu, Xiao-Peng Mei

**Affiliations:** 1 Department of Anesthesiology, School of Stomatology, Fourth Military Medical University, Xi'an, China; 2 Department of Prosthodontics, School of Stomatology, Fourth Military Medical University, Xi'an, China; 3 Department of Orthodontics, School of Stomatology, Fourth Military Medical University, Xi'an, China; University of Medicine & Dentistry of NJ - New Jersey Medical School, United States of America

## Abstract

Neuropathic pain is an intractable clinical problem. Drug treatments such as tramadol have been reported to effectively decrease neuropathic pain by inhibiting the activity of nociceptive neurons. It has also been reported that modulating glial activation could also prevent or reverse neuropathic pain via the administration of a glial modulator or inhibitor, such as propentofylline. Thus far, there has been no clinical strategy incorporating both neuronal and glial participation for treating neuropathic pain. Therefore, the present research study was designed to assess whether coadministration of tramadol and propentofylline, as neuronal and glial activation inhibitors, respectively, would exert a synergistic effect on the reduction of rat spinal nerve ligation (SNL)-induced neuropathic pain. Rats underwent SNL surgery to induce neuropathic pain. Pain behavioral tests were conducted to ascertain the effect of drugs on SNL-induced mechanical allodynia with von-Frey hairs. Proinflammatory factor interleukin-1β (IL-1β) expression was also detected by Real-time RT-PCR. Intrathecal tramadol and propentofylline administered alone relieved SNL-induced mechanical allodynia in a dose-dependent manner. Tramadol and propentofylline coadministration exerted a more potent effect in a synergistic and dose dependent manner than the intrathecal administration of either drug alone. Real-time RT-PCR demonstrated IL-1β up-expression in the ipsilateral spinal dorsal horn after the lesion, which was significantly decreased by tramadol and propentofylline coadministration. Inhibiting proinflammatory factor IL-1β contributed to the synergistic effects of tramadol and propentofylline coadministration on rat peripheral nerve injury-induced neuropathic pain. Thus, our study provided a rationale for utilizing a novel strategy for treating neuropathic pain by blocking the proinflammatory factor related pathways in the central nervous system.

## Background

Neuropathic pain is a refractory problem for clinical treatment and laboratory research. Multiple mechanisms are involved in the initiation and maintenance of peripheral nerve injury-induced neuropathic pain, such as hyperexcitable primary afferents, abnormal plasticity in the spinal dorsal horn, and aberrant neuronal-glial interactions [1].

Traditionally, the treatment of neuropathic pain has involved the inhibition of spinal nociceptive neuronal activation after nerve injury with drugs such as morphine, gabapentin and tramadol. Accumulating evidence suggested that tramadol is useful for treating neuropathic pain as a neuronal activation inhibitor [2], [3]. However, consistent with the clinical concept of balanced or associative manner, the use a combination of analgesics was postulated to provide better pain control for patients with neuropathic pain [4]. Therefore, the coadministration of tramadol with some other analgesic should theoretically exert more potent pain relief [2], [5]. Prior studies of the coadministration of drugs have focused solely on neuronal mechanisms and neglected glial cell participation, which is another key factor in neuropathic pain development.

Previous reports have suggested that spinal glial activation is required and sufficient for neuropathic pain development [6], [7]. Glia contribute to neuronal excitability by releasing neurotransmitters, extracellular signaling molecules, chemokines, and cytokines, as well as reuptaking neurotransmitters from synaptic clefts, thus coordinating activity among neuronal networks [1], [8]. Previous reports have also indicated that spinal glia (especially microglia and astrocytes) are key factors in the initiation and maintenance of neuropathic pain processing [6], [7]. Therefore, inhibiting spinal glial activation is another option for treating neuropathic pain. Propentofylline is a unique methylxanthine with clear cyclic AMP, phosphodiesterase, and adenosine actions, including enhanced synaptic adenosine signaling [9]. Propentofylline has been shown to produce profound neuroprotective, antiproliferative, and anti-inflammatory effects on stroke, opioid tolerance, and acute and chronic pain [10], [11]. These effects depend on modulating spinal glial activity and proliferation, which consequently lower the expression of proinflammatory cytokines and chemokines, as well as neuronal activity [10]. Recent studies have demonstrated that spinal neuronal and glial “cross-talk” are the most important mechanisms underlying the development of neuropathic pain [1], [12]. Therefore, the combination of a neuronal inhibitor and glial modulator may improve the treatment of neuropathic pain by interrupting the positive feedback interaction between neurons and glia [8].

The hypothesis of the present study was that the combination of tramadol and propentofylline might exert a synergistic effect on decreasing spinal nerve ligation (SNL)-induced neuropathic pain and lead to an improved and novel strategy for the clinical treatment of patients with neuropathic pain.

In this study, the antiallodynic effect of drug combinations were evaluated with a pain behavioral test, while the experimental ED50 and the theoretical ED50 of drug combinations were determined and analyzed with isobolographic analyses to verify whether there were any synergistic effects of the coadministration of drugs on SNL-induced neuropathic pain.

## Materials and Methods

### Experimental animals

Male *Sprague–Dawley* rats (170–190 g) were kept in plastic cages with food and water available. The ambient temperature was about 22–25°C with a 12∶12 h light/dark cycle. All experimental procedures received prior approval (No. 12015) from the Animal Use and Care Committee for Research and Education of the Fourth Military Medical University (Xi'an, China), and the ethical guidelines to investigate experimental pain in conscious animals [13]. All efforts were made to minimize animal suffering and to reduce the number of animals used.

### Surgical procedures

Intrathecal implantation was carried out by inserting polyethylene (PE) tubing to inject drugs directly into the subarachnoid space of the lumbar enlargement. Briefly, a midline incision (3 cm) was made at the back of the rat from the level of the 3rd thoracic vertebrae to the lower back, under pentobarbital anesthesia (45 mg kg-1, i.p.). A pre-measured length of PE-10 tubing (I.D. 0.28 mm and O.D. 0.61 mm) was passed caudally from the T8 to the L3 level of the spinal cord, and 2 cm of the free ending was left exposed in the upper thoracic region. Rats were allowed to recover for a 3–5 d before further use. Only the animals judged as neurologically normal and that showed complete paralysis of the tail and bilateral hind legs after administration of 2% lidocaine (10 µl) through the intrathecal catheter were used for the following experiments.

The animal neuropathic pain model was conducted with spinal nerve ligation under pentobarbital anesthesia (45 mg/kg, *i.p.*). The L6 vertebra left transverse process of animal was first removed to expose the L4 and L5 spinal nerves. The L5 spinal nerve was then carefully isolated and tightly ligated with 6-0 silk thread [14]. The surgical procedure for the sham group was identical to that of the SNL group, except that the spinal nerve was not ligated.

### Mechanical allodynia test

Animals were habituated to the testing environment for 3 d before baseline testing, and then were placed under inverted plastic boxes (30×30×50 cm^3^) on an elevated mesh floor and to allow habituation for 30 min before the threshold testing. Briefly, a logarithmic series of 8 calibrated Semmes-Weinstein monofilaments (von-Frey hairs; Stoelting, Kiel, WI, USA) were applied to the ipsilateral hindpaws to determine the stimulus intensity threshold stiffness required to elicit a paw withdrawal response [15]. Log stiffness of the hairs is determined by log10 (milligrams×10) [16]. The 8 filaments had the following log-stiffness values (value in grams is given in parentheses): 4.17 (1479 mg), 4.31 (2041 mg), 4.56 (3630 mg), 4.74 (5495 mg), 4.93 (8511 mg), 5.07 (11749 mg), 5.18 (15136 mg), and 5.46 (28840 mg). The range of monofilaments (1.479–28.840 gm) produced a logarithmically graded slope when interpolating a 50% response threshold of stimulus intensity (expressed as log10 (milligrams×10)) [17]. Assessments were made before SNL surgery for baseline value. Then the behavioral tests were performed on post operative day (POD) 7 after drugs administration from POD 0 to POD 7. The behavioral responses were used to calculate the 50% paw withdrawal threshold, by fitting a Gaussian integral psychometric function using a maximum-likelihood fitting method, as described in detail previously [16]. This fitting method allowed parametric statistical analysis. The percentage of the antiallodynia was calculated according to the following equation [18]: % Antiallodynia = 100−100× (baseline of SNL-Drug – post SNL-Drug)/(baseline of SNL-Saline – post SNL-Saline). All behavioral tests were performed in a double-blind manner.

### Real-time reverse transcription polymerase chain reaction (RT-PCR)

Rats were anesthetized with sodium pentobarbital (60 mg/kg, i.p.) and killed. As described previously [19], the ipsilateral L5 spinal dorsal horn was rapidly harvested and total RNA was extracted with Trizol (GIBCO/BRL Life Technologies Inc., Grand Island, NY, USA). Complementary DNA (cDNA) was synthesized with oligo (dT)_12–18_ using Superscript™ III Reverse Transcriptase for RT-PCR (Invitrogen, Carlsbad, CA, USA). The primers used in the present study were presented in Table 1. Equal amounts of RNA (1 µg) were used to prepare cDNA using the SYBR® Premix Ex Taq™ (Takara, Tokyo, Japan) and analyzed by real-time PCR in a detection system (Applied Biosystems, Foster City, CA, USA). The amplification protocol was: 3 min at 95°C, followed by 45 cycles of 10 s at 95°C for denaturation and 45 s at 60°C for annealing and extension. All experiments were repeated twice and, in each experiment, PCR reactions were done in triplicate. Target cDNA quantities were estimated from the threshold amplification cycle number (C_t_) using Sequence Detection System software (Applied Biosystems). A ΔCt value was calculated for each sample by subtracting their Ct value from the Ct value for the corresponding GAPDH to normalize the differences in cDNA aliquots. Each cDNA quantity was then calculated with the following formula: 2^ΔCt^. GAPDH was served as an endogenous internal standard control for variations in RT-PCR efficiency.

**Table 1:** Primers sequence for the rat genes characterized in present experiment.

Genes	Primers	Sequences	Accession number
IL-1beta	Forward primer	5′-TGCTGATGTACCAGTTGGGG-3′	NM031512
	Reverse primer	5′-CTCCATGAGCTTTGTACAAG-3′	
GAPDH	Forward primer	5′-CCCCCAATGTATCCGTTGTG-3′	NM01008
	Reverse primer	5′-TAGCCCAGGATGCCCTTTAGT-3′	

### Drugs application

Tramadol hydrochloride and propentofylline (Sigma) were dissolved and diluted with preservative-free normal saline solution for administration. Normal saline (0.9%) was used as the negative control. Tramadol was injected in doses of 3, 10 and 30 µg/rat, and propentofylline in doses of 0.1, 0.5 and 2.5 µg/rat. The dosages of each drug applied in the present study are based on our pilot study and previous reports [10], [20]. The dose-effect curve was constructed and the experimental points fitted using least-square linear regression. Then, the ED50 (50% antiallodynia) of each drug was calculated [21]. To assess interaction between drugs, tramadol and propentofylline were administered in fixed ratio combination (tramadol ED50/2 + propentofylline ED50/2 ug/rat; tramadol ED50/4 + propentofylline ED50/4 ug/rat and tramadol ED50/8 + propentofylline ED50/8 ug/rat). Drugs and saline (10 µl each) were injected intrathecally over 30 s, followed by a 10 µl flush of normal saline.

### Deficit of motor function evaluation

In order to evaluate whether the drugs applied in the present study could affect motor function, which might influence the behavioral results, we performed rotarod tests on intrathecal drug administered but operation- and behavioral observation-free rats [22]. Rats without previous exposures to the rotarod test were placed on the Ugo Basile 7650 Rotarod accelerator treadmill (Ugo Basile, Varese, Italy) set at the minimal speed for training sessions of 1–2 min at intervals of 30–60 min. After this learning period, the animals were placed on to the rotarod at a constant speed of 25 *RPM*. As the animal took a grip of the drum, the accelerator mode was selected on the treadmill, *i.e.* the rotation rate of the drum was increased linearly at 20 *RPM*. Thereafter, the time was measured from the start of the acceleration period until the rat fell off the drum. The cut-off time was 30 s. Each rat was tested 30 min before drug administration as control performance and then once a day for 7 day during the drug administration. The time that the animal remained on the rotarod was recorded and expressed as a percentage of that animal's own mean control performance.

### Statistical analysis

All data were collected by researchers blinded to the surgery and reagents used. Data from the von-Frey test were presented as mean ± SD and analyzed as the interpolated 50% threshold (absolute threshold) in log base 10 of stimulus intensity (monofilament stiffness in milligrams×10). Repeated measures ANOVA (with Bonferroni confidence interval adjustment) was used and conducted for analyzing. Data from the rotarod test were presented as mean ± SD. Repeated measures ANOVA (with Bonferroni confidence interval adjustment) was used and conducted for analyzing.

Isobolographic analysis was used for evaluating the interaction after drugs coadministration [18], [21]. In brief, ED50 of each drug was calculated by regression analysis. A combination of the two drugs was administered in a constant dose ratio based on ED50 values (tramadol ED50/2 + propentofylline ED50/2 ug/rat; tramadol ED50/4 + propentofylline ED50/4 ug/rat and tramadol ED50/8 + propentofylline ED50/8 ug/rat). For drugs combination, the theoretic ED50 is tramadol ED50/2 + propentofylline ED50/2. Experimental values of drugs combination from fixed ratio designed studies were also analyzed using regression, and then the experimental ED50 value of drugs combination was calculated (50% antiallodynia in SNL-induced mechanical allodynia). The statistical significance between the theoretical ED50 and experimental ED50 of drugs combination was evaluated with Student's t test. An experimental ED50 significantly less than the theoretical ED50 was considered to indicate a synergistic interaction between tramadol and propentofylline.

All statistical analyses were performed using SPSS® version 16.0 software (SPSS Inc., Chicago, IL, USA). *P*<0.05 was considered statistically significant.

### Experimental protocols

Experimental rats received intrathecal intubation and were allowed to recover for 3–5 d before further use. The baseline value of the behavioral test was carried out on the SNL or sham surgical day prior to the beginning of drugs administration. SNL or sham surgical procedure was carried out after drugs administration on the post operative day (POD) 0. Drugs were applied for 8 d from POD 0 to POD 7. Then the behavioral tests were performed on POD 7 after drugs administration. Finally, all rats were sacrificed for RT-PCR after the behavioral test on POD 7.

## Results

### Intrathecal tramadol alone and propentofylline alone attenuated SNL-induced mechanical allodynia in a dose-dependent manner

To detect the effects of intrathecal tramadol and propentofylline administered alone on SNL-induced neuropathic pain, these two reagents were injected individually at three different concentrations for 8 days, from the SNL operation day to post-operative day (POD) 7. The changes of the mechanical pain threshold were observed on POD 7.

**Figure 1:**
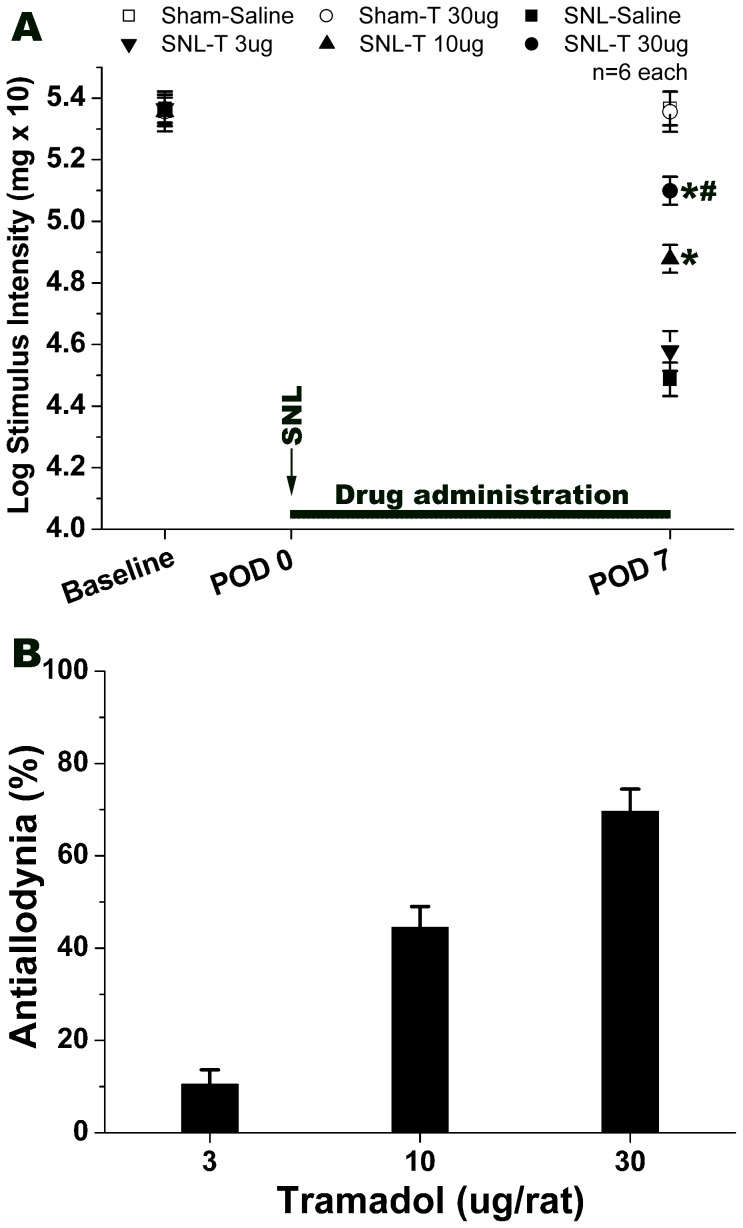
Effect of intrathecal tramadol on spinal nerve ligation (SNL)-induced neuropathic pain. Graph A shows a dose-dependent effect of intrathecal tramadol on SNL-induced mechanical allodynia. Intrathecal tramadol (10 or 30 µg/rat) significantly elevated the pain threshold, whereas, intrathecal tramadol 3 µg/rat didn't show apparent effect. Graph B means the percentage of anti-allodynia of the maximum possible effect. The Y-axis is the percentage of the antiallodynia after drug administration. % Antiallodynia = 100−100×(baseline of SNL-Drug – post SNL-Drug)/(baseline of SNL-Saline – post SNL-Saline). *P<0.05, compared with that of SNL-Saline. ^#^ *P*<0.05, compared with that of SNL-T 10 µg group. 6 rats in each group. POD: post operative day, T: tramadol.

Intrathecal application of 3 µg tramadol from POD 0 to POD 7 had no obvious effect on SNL-induced mechanical allodynia (Fig. 1A). Intrathecal tramadol at 10 µg elevated the pain threshold significantly compared to that of SNL-Saline group (Fig. 1A, *P*<0.05). A higher dose of tramadol (30 µg) relieved SNL-induced mechanical allodynia apparently on POD 7 (Fig. 1A, *P*<0.05, compared to that of SNL-Saline control). Furthermore, 30 µg of tramadol exhibited a stronger antiallodynic effect than 10 µg of tramadol (Fig. 1A, *P*<0.05). However, the pain threshold of sham rats was not affected by intrathecal tramadol even at a 30 µg dose. Intrathecal tramadol (3, 10 or 30 µg) demonstrated an effective and reliable antiallodynic effect in a dose-dependent manner on SNL-induced neuropathic mechanical allodynia (Fig. 1B). Additionally, the ED50 dose (50% antiallodynia) of intrathecal tramadol was evaluated by regression analysis (Table 2).

**Table 2:** Dose used in the study of the interaction between tramadol and propentofylline after intrathecal administration.

Tramadol in the combination		Propentofylline in the combination		Total dose	
Dose (ug)	Antiallodynia (%)	Dose (ug)	Antiallodynia (%)	Dose (ug)	Antiallodynia (%)
					(Theoretically)
13.41	50.0 (ED50)	0.99	50.0 (ED50)		
6.70	25.0 (ED50/2)	0.50	25.0 (ED50/2)	7.20	50.0 (ED50)
3.35	12.5 (ED50/4)	0.25	12.5 (ED50/4)	3.60	25.0 (ED50/2)
1.68	6.25 (ED50/8)	0.12	6.25 (ED50/8)	1.80	12.5 (ED50/4)

**Figure 2:**
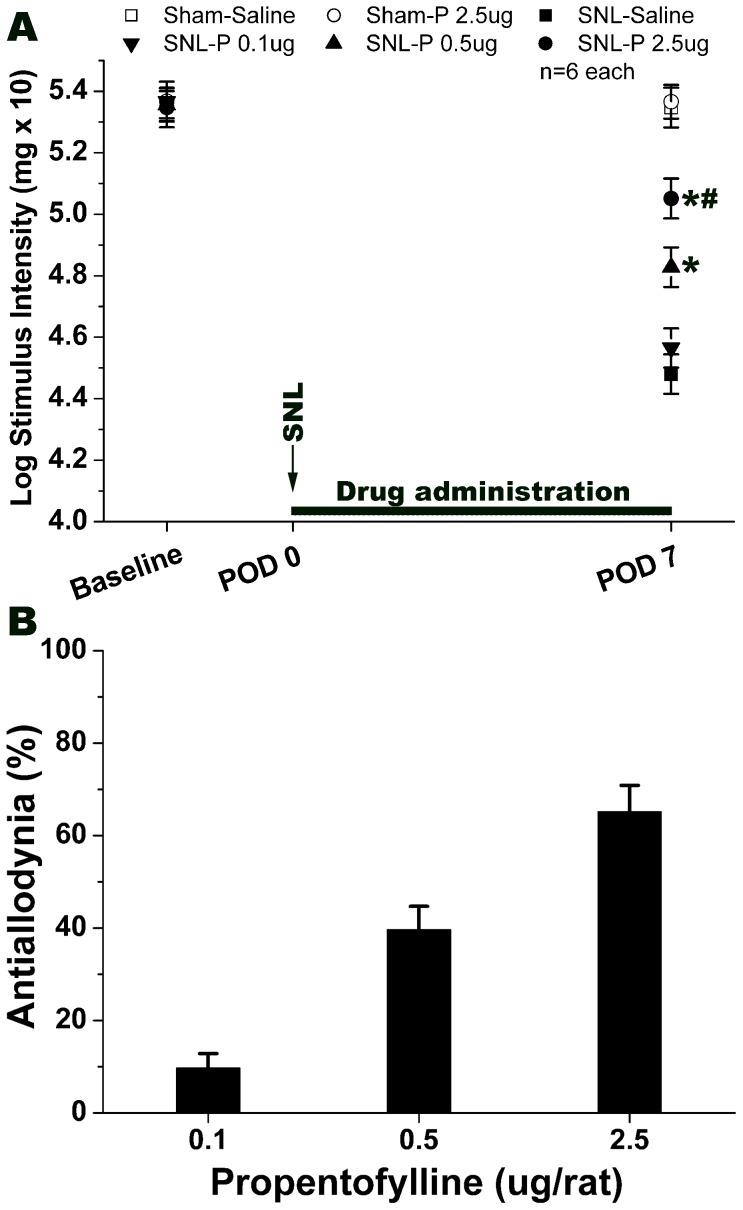
Effect of intrathecal propentofylline on SNL-induced neuropathic pain. Graph A shows a dose-dependent effect of intrathecal propentofylline on SNL-induced neuropathic pain. Intrathecal propentofylline (0.5 or 2.5 µg/rat) markedly raised the pain threshold, whereas, intrathecal propentofylline 0.1 µg/rat didn't show obvious effect on SNL-induced allodynia. Graph B means the percentage of anti-allodynia of the maximum possible effect. The Y-axis is the percentage of the antiallodynia after drug administration. % Antiallodynia = 100−100×(baseline of SNL-Drug – post SNL-Drug)/(baseline of SNL-Saline – post SNL-Saline). *P<0.05, compared with that of SNL-Saline. ^#^ *P*<0.05, compared with that of SNL-P 0.5 µg group. 6 rats in each group. P: propentofylline.

Intrathecal 0.1 µg of propentofylline did not produce antiallodynic effects on SNL-induced neuropathic pain. Application of propentofylline at 0.5 µg could attenuate SNL-induced neuropathic pain on POD 7 (Fig. 2A, *P*<0.05, compared with that of SNL-Saline group). A higher dose of propentofylline (2.5 µg) significantly suppressed SNL-induced mechanical allodynia compared to the SNL-Saline group (Fig. 2A, *P*<0.05). Furthermore, the antiallodynic effect of 2.5 µg of propentofylline was greater than that of 0.5 µg of propentofylline (Fig. 2A, *P*<0.05). However, even the application of 2.5 µg propentofylline did not change the pain threshold of the sham-operated rats. Intrathecal propentofylline (0.1, 0.5 or 2.5 µg) exerted an antiallodynic effect in a dose-dependent manner on SNL-induced neuropathic pain (Fig. 2B). Finally, the ED50 dose (50% antiallodynia) of intrathecal propentofylline was calculated by regression analysis (Table 2).

### Effects of tramadol and propentofylline coadministration on SNL-induced neuropathic pain

To investigate the effect of drug combinations on SNL-induced mechanical allodynia, tramadol and propentofylline were coapplied in a constant dose ratio based on ED50 values (tramadol ED50/2 + propentofylline ED50/2, tramadol ED50/4 + propentofylline ED50/4 and tramadol ED50/8 + propentofylline ED50/8 ug/rat). The doses in combination were 7.2, 3.6 and 1.8 µg (Table 2).

**Figure 3:**
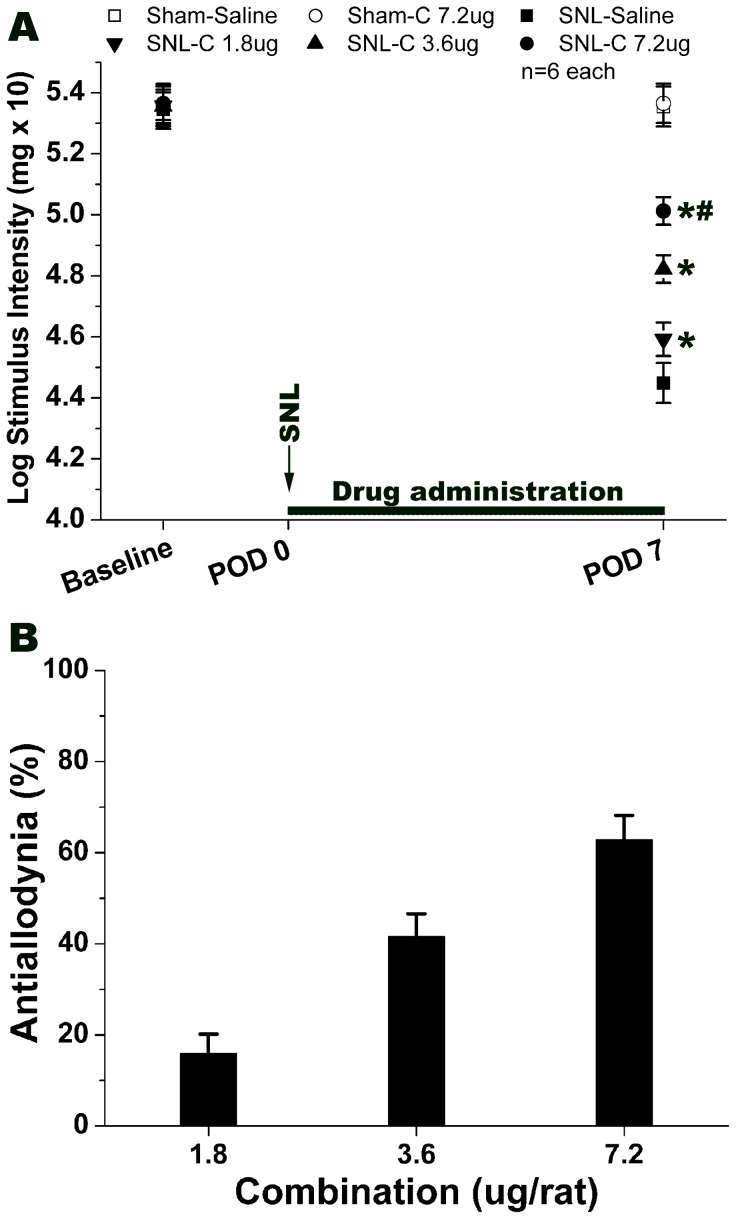
Effect of tramadol and propentofylline intrathecal coadministration on SNL-induced neuropathic pain. Graph A shows a dose-dependent effect of drugs coadministration on SNL-induced mechanical allodynia. Coadministration of 7.2 µg/rat (6.7 µg/rat tramadol and 0.5 µg/rat propentofylline) remarkably reversed the mechanical allodynia. Combination of 3.6 µg/rat (3.35 µg/rat tramadol and 0.25 µg/rat propentofylline) also effectively elevated the pain threshold. Moreover, intrathecal 1.8 µg/rat (1.68 µg/rat tramadol and 0.12 µg/rat propentofylline) could still relief the mechanical allodynia. Graph B means the percentage of anti-allodynia of the maximum possible effect. The Y-axis is the percentage of the antiallodynia after drug administration. % Antiallodynia = 100−100×(baseline of SNL-Drug – post SNL-Drug)/(baseline of SNL-Saline – post SNL-Saline). *P<0.05, compared with that of SNL-Saline. ^#^ *P*<0.05, compared with that of SNL-C 3.6 µg group. 6 rats in each group. C: coadministration.

Coadministration of 1.8 µg of the drugs relieved SNL-induced neuropathic pain. Drug combinations of 3.6 µg significantly elevated the pain threshold of the nerve injury animals compared to the SNL-Saline group (Fig. 3A, *P*<0.05). Furthermore, the injection of 7.2 µg of the drugs in combination demonstrated a marked effect on SNL-induced mechanical allodynia compared to that of SNL-Saline group (Fig. 3A, *P*<0.05). However, 7.2 µg of drug in combination had no effect on sham operative rats. Tramadol and propentofylline in combination generated antiallodynic effects on SNL-induced neuropathic pain in a dose-dependent manner (Fig. 3B). The experimental ED50 dose (50% antiallodynia) of the drugs in combination was calculated by regression analysis.

**Figure 4:**
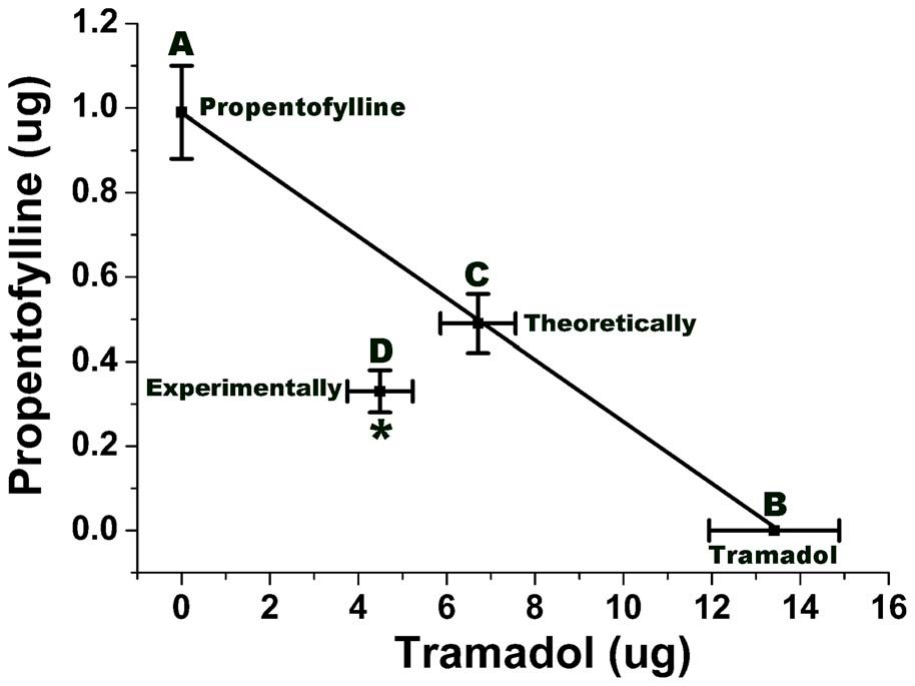
Isobologram of drugs combination shows the synergistic effect of intrathecal tramadol and propentofylline coadministration on SNL-induced neuropathic pain. Graph A, B means the ED50 of intrathecal propentofylline or tramadol respectively. The oblique line between A and B is the theoretic additive effect line of tramadol and propentofylline coadministration. Graph C, in the middle of the line, is the theoretical ED50 of drugs combination, which is calculated from the individual drug ED50. Graph D, far below the line, is the experimental ED50 of drugs combination, which is actually observed after drugs coadministration. The experimental ED50 point lies far below the additive line, suggesting a significant synergistic effect of drugs coadministration. *P<0.05, compared with that of theoretical ED50.

To confirm whether tramadol and propentofylline coadministration would exert a synergistic effect on SNL-induced neuropathic pain, the isobolographic analysis compared the theoretical ED50 with the experimental ED50 of the drugs in combination (Fig. 4). The theoretical additive line indicated that all points of tramadol and propentofylline combinations in this line produced an effect of theoretical 50% antiallodynia (Theoretical ED50) according to an additive interaction (Fig. 4). The experimental 50% antiallodynia (Experimental ED50) value of tramadol and propentofylline coadministration was located below the theoretical additive line, suggesting a synergistic effect of tramadol and propentofylline coadministration on SNL-induced neuropathic pain. The significant difference between the experimental ED50 and the theoretical ED50 was verified by Student's t test (Fig. 4, *P*<0.05).

### Tramadol and propentofylline coadministration inhibited SNL-induced spinal dorsal horn IL-1β expression

**Figure 5:**
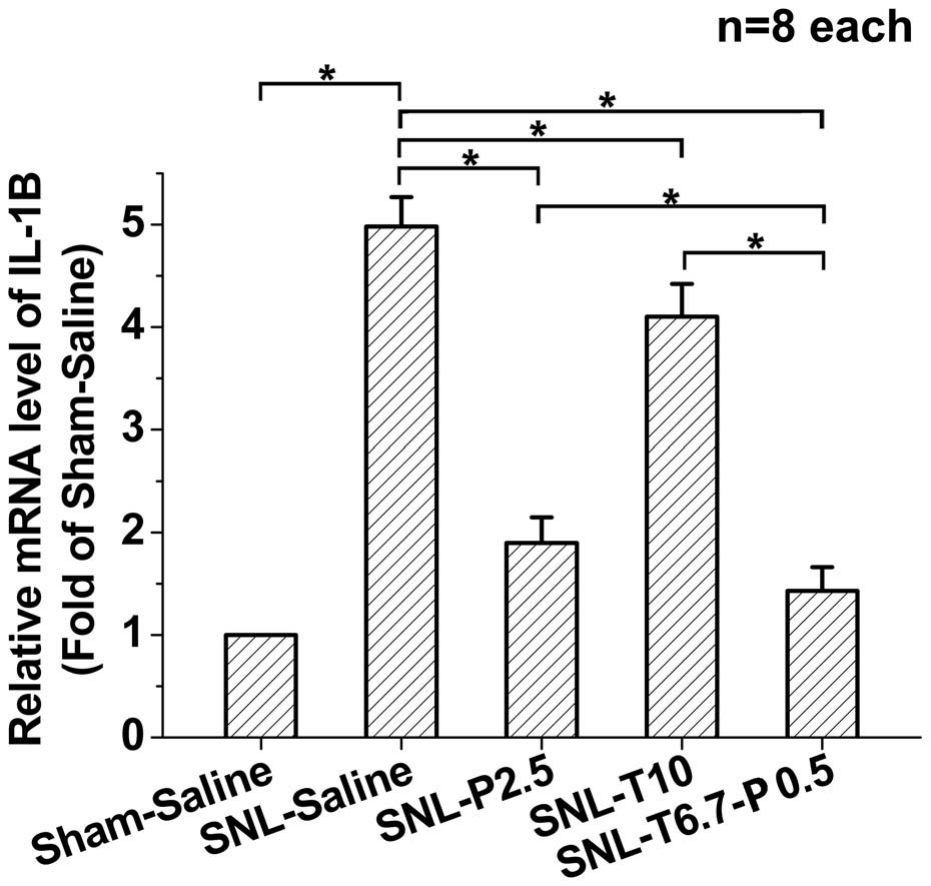
Effects of drugs application on SNL-induced IL-1β expression. Different drugs were injected to detect the effects on SNL-induced IL-1β expression. *P<0.05. 8 rats in each group. C: coadministration.

The expression of spinal dorsal horn IL-1β was up-regulated in SNL-Saline group compared to that of the Sham-Saline group (Fig. 5). The intrathecal administration of propentofylline 2.5 µg (SNL-P2.5) depressed IL-1β expression significantly compared to the SNL-Saline group (Fig. 5). Moreover, 10 µg tramadol (SNL-T10) also suppressed the SNL-induced IL-1β expression compared to the SNL-Saline group (Fig. 5). Additionally, the combination of 6.7 µg tramadol with 0.5 µg propentofylline (SNL-T6.7-P0.5) exerted a more powerful inhibiting effect on SNL-induced IL-1β expression than that of tramadol at 10 µg or propentofylline at 2.5 µg administered alone (Fig. 5).

### Effects of drugs on motor functions indicated by the rotarod test

**Figure 6:**
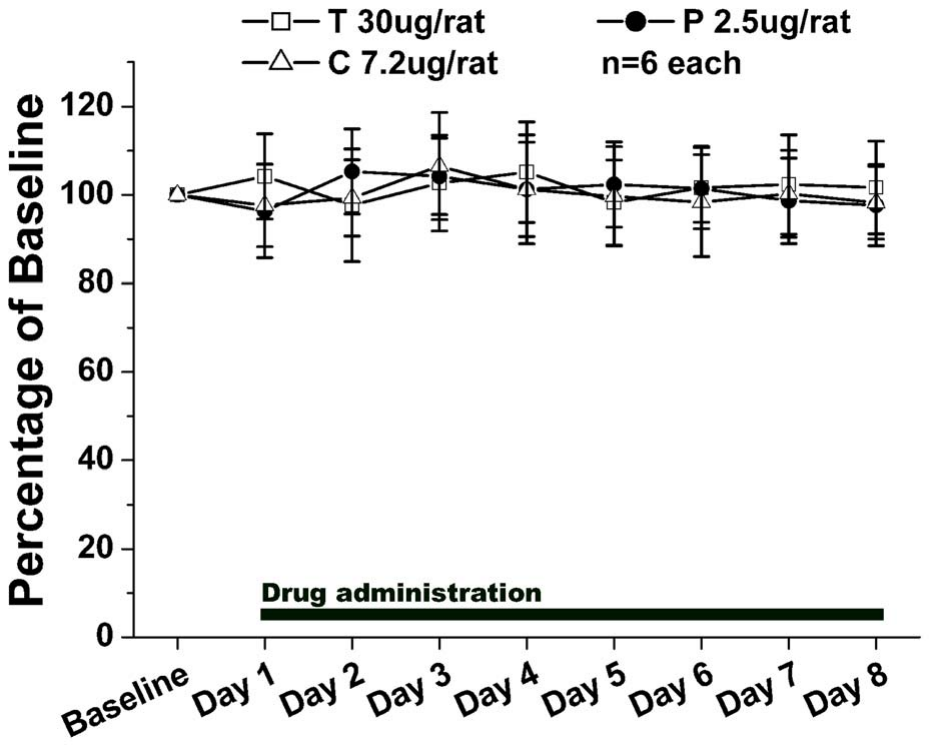
Effects of drugs on motor performance of rats in the rotarod test. After a baseline response had been obtained, tramadol 30 µg, propentofylline 2.5 µg and combination 7.2 µg were administered intrathecally and rotarod test was performed once a day for 8 d. Compared with that of the baseline response, there was no statistical differences could be detected from rotarod test after drugs intrathecal administration. 6 rats in each group.

Nociceptive behavioral results can be altered by motor dysfunction. To assess whether the drugs (each in high dose: tramadol 30 µg, propentofylline 2.5 µg or drugs in combination 7.2 µg) used in the present study would impair motor functions, 18 otherwise experiment-free rats were assessed with the rotarod test. Repeated administrations of drugs (tramadol 30 µg, propentofylline 2.5 µg or drugs in combination 7.2 µg) did not affect the motor performance of rats compared to their own performance at baseline (Fig. 6).

## Discussion

Both tramadol and propentofylline are reported to be useful for treating neuropathic pain [23], [24]. However, it is not known whether tramadol and propentofylline would have an interactive effect when coadministered. The present study indicated that the intrathecal administration of tramadol alone and propentofylline alone relieved SNL-induced mechanical allodynia in a dose-dependent manner. Coadministration of tramadol and propentofylline exhibited synergistic antiallodynic effects on SNL-induced neuropathic pain, also in a dose-dependent manner. Real-time RT-PCR demonstrated that tramadol and propentofylline coadministration significantly decreased SNL-induced ipsilateral spinal dorsal horn IL-1β up-expression compared to each drug given alone. Moreover, drug administration did not affect the motor function of the rats. Taken together, the present study suggests that coadministration of tramadol and propentofylline could exert synergistic antiallodynic effects on SNL-induced neuropathic pain by inhibiting SNL-induced IL-1β related pathways. These finding support a novel strategy for treating peripheral nerve injury-induced neuropathic pain.

### Tramadol for neuropathic pain, as a neuronal activation inhibitor

Both clinical and basic research have demonstrated that intrathecal tramadol is effective for treating neuropathic pain [2], [25]. Compatible with previous studies, the present work indicated that intrathecal tramadol could attenuate SNL-induced neuropathic pain in a dose-dependent manner. Tramadol is an opioid receptor agonist that acts on the mu opioid receptor and suppresses pain-induced spinal dorsal horn neuronal activation [26]. Peripheral nerve injury-induced neuropathic pain can be treated by opioid receptor agonists such as morphine and tramadol [23], [27]. Therefore, inhibiting neuronal activation by suppressing mu opioid receptor activity could be a potential mechanism by which intrathecal tramadol acts on SNL-induced neuropathic pain. In addition, tramadol has been reported to inhibit serotonin and norepinephrine reuptake at the spinal level [26], [28]. Serotonin and norepinephrine are known as two important neurotransmitters released from the descending inhibition system and can exert analgesic effects at the spinal level [29], [30]. Therefore, blocking the reuptake of these two neurotransmitters may be another antiallodynic mechanism of tramadol supported in the present study.

### Propentofylline for neuropathic pain, as a glial activation inhibitor

A previous study concluded that spinal glial cells play a pivotal role in the initiation and maintenance of nerve injury-induced neuropathic pain [31]. Propentofylline, a glial cell modulator, was reported to be effective for treating neuropathic pain induced by peripheral nerve injury and spinal cord injury [10], [11], [24], [32], [33]. Consistent with previous research [10], [24], [33], our present study confirmed that intrathecal propentofylline exerted obvious antiallodynic effects on SNL-induced neuropathic pain. A possible mechanism of action is that intrathecal propentofylline could suppress aberrant glial activation in peripheral nerve injury-induced neuropathic pain by modulating glial glutamate transporter functioning. [11]. Furthermore, it has been reported that intrathecal treatment with propentofylline significantly attenuated astrocytic and microglial activation and significantly prevented the decrease of glutamic acid decarboxylase (GAD)(65) expression in both sides of the lumbar dorsal horn following spinal cord injury. This result suggests that propentofylline modulates glial activation and GABAergic inhibitory tone by modulation of GAD(65) protein expression [24]. Therefore, previous results support our present study in which intrathecal propentofylline produced a potent and consistent antiallodynia in models of neuropathic pain. This effect could be the result of inhibition of aberrant spinal glial activation and the modulation of molecular expression.

### Coadministration of the drugs

The modern clinical concept of balanced or associative manner proposes that the coadministration of analgesics or the combination of other treatments provides better pain relief and minimizes side effects compared to a single treatment alone [4]. Because it has been reported that not only neuronal but also glial activation contributes to the initiation and maintenance of nerve-injury-induced neuropathic pain [1], [31], the present study combined tramadol with propentofylline to propose a novel strategy for treating neuropathic pain.

Our study indicates that the coadministration of tramadol and propentofylline exerted some synergistic antiallodynic effects on SNL-induced neuropathic pain in a dose-dependent manner. This antiallodynic effect of the coadministration of drugs was stronger than that of intrathecal tramadol or propentofylline alone. The experimental ED50 of drug coadministration was much lower than the theoretical ED50, suggesting that the combination of tramadol with propentofylline may be an effective way to alleviate SNL-induced mechanical allodynia.

Glial cells represent the largest cell population in the central nervous system (CNS) and contribute to neuropathic-pain processing by releasing a number of glial and neuronal signaling molecules, such as cytokines and chemokines [1], [34], [35]. Evidence suggests that the activation of glial cells leads to pro-inflammatory responses with pathological effects, such as neuronal hyperexcitability, neurotoxicity and chronic inflammation [34]. SNL induced both spinal microglial and astrocytic activation in a quick and long lasting response pattern [31]. Activated glial cells show a stereotypic progressive series of changes in morphology, gene expression and function, and they release various chemical mediators, including proinflammatory cytokines, complement components and other substances that facilitate pain transmission. These activated glial cells may be involved in the development of neuropathic pain by modulating spinal dorsal horn neuron activity [7], [36], [37]. Recent evidence has supported the hypothesis that IL-1β released from activated glial cells contributes to neuronal activation [38], [39].

Spinal nerve injury (SNI)-induced mechanical allodynia could be reversed by administering an intrathecal glial metabolic inhibitor (fluorocitrate). Furthermore, mitogen-activated kinases, inhibitors of p38, have been shown to prevent SNI-induced mechanical allodynia by inhibiting proinflammatory cytokine IL-1β production and signaling. Moreover, SNI-induced mechanical allodynia was prevented by intrathecal proinflammatory cytokine antagonists specific for IL-1β [16]. These results provide solid evidence that peripheral nerve injury (SNI or SNL)-induced spinal dorsal horn glial cells and the MAPK p38 and proinflammatory cytokine IL-1β pathways contribute to the development of neuropathic pain. The present study demonstrates that intrathecal tramadol and propentofylline depress SNL-induced spinal dorsal horn IL-1β production, which contributes to the synergistic effects of tramadol and propentofylline coadministration on the development of SNL-induced neuropathic pain.

IL-1β may be a potential target in the management of neuropathic pain after nerve injury. A previous study has demonstrated that the mRNA and protein levels of IL-1β are rapidly up-regulated after sciatic nerve injury. Mice lacking IL-1β or IL-1 type 1 receptor (IL-1R1) exhibited reduced mechanical allodynia compared with wild-type littermates after nerve injury. Microinjecting recombinant IL-1β at the site of sciatic nerve injury in IL-1β knock-out mice restored mechanical pain thresholds to levels observed in injured wild-type mice. These results suggest that targeting specific IL-1β-dependent responses has potential as a therapeutic strategy for treatment of neuropathic pain after peripheral nerve injury [40].

Taken together, neuronal input generated by injury-induced neuronal hyperexcitability in the pain-processing circuitry, which activated the glia and led to the release of cytokines and other chemical mediators from glial cells. Cytokines released from the glia triggered neurons to facilitate central sensitization and promote pain processing. This scenario suggests a model of glial–cytokine–neuronal interaction underlying the mechanisms of neuropathic pain [39]. Thus, inhibiting glial activation could modulate neuronal activation. Therefore, the pharmacological combination of glial activation modulator/inhibitor with a neuronal activation inhibitor/modulator could exert synergistic effects and represent a potential novel strategy for treating neuropathic pain. Accordingly, our present study indicated that combined propentofylline with tramadol synergistically alleviated SNL-induced neuropathic pain.

Additionally, peripheral nerve injury-induced spinal neuronal activation involves the synthesis of various transmitters, excitatory amino acids, ATP and chemokines [41], [42]. Fractalkine, also known as CX3CL1, a type of chemokine, has been suggested to mediate signals between neuron and glia. Fractalkine has been synthesized and released by spinal neurons, and the receptor CX3CR1 has been expressed in spinal microglia after peripheral nerve injury [43], [44]. This neuron-to-glia interaction contributes to the development of nerve injury-induced neuropathic pain. Studies also indicate that ATP is released from injured primary afferents and spinal dorsal horn neurons and induces spinal glial activation after binding to P2X4 receptor in glia [8], [45].

## Conclusions

The present results indicate that both intrathecal tramadol (a neuronal activity inhibitor) and propentofylline (a glial activation modulator) administered alone relieve peripheral nerve injury-induced neuropathic pain effectively. Furthermore, the coadministration of tramadol and propentofylline exerts a synergistic effect on neuropathic pain reduction by inhibiting proinflammatory factor IL-1β activity. In accordance with a recent report [1] concerning the modern concept of the spinal mechanisms of neuropathic pain development, our previous study concurred that there is a positive feedback loop between neuronal and glial activation [19]. Therefore, our present study supports a novel strategy for treating peripheral nerve injury-induced neuropathic pain with a combination of glial activation modulator/inhibitor and a neuronal activation inhibitor/modulator.
